# Risk factors and primary indications for cesarean section in women with gestational diabetes mellitus according to Robson classification

**DOI:** 10.1016/j.clinsp.2025.100705

**Published:** 2025-06-14

**Authors:** Ana Paula Veiga de Oliveira, Tatiana Assunção Zaccara, Marcela Del Carlo Bernardi, Cristiane de Freitas Paganoti, Fernanda Cristina Ferreira Mikami, Rossana Pulcineli Vieira Francisco, Rafaela Alkmin da Costa

**Affiliations:** a Disciplina de Obstetrícia, Departamento de Obstetrícia e Ginecologia, Faculdade de Medicina da Universidade de São Paulo (FMUSP), Universidade de São Paulo, São Paulo, Brazil; b Divisão de Obstetricia, Instituto Central, Hospital das Clínicas da Faculdade de Medicina da Universidade de São Paulo (HCFMUSP), Faculdade de Medicina, Universidade de São Paulo, São Paulo, Brazil

**Keywords:** Gestational diabetes, Cesarean section, Robson classification

## Abstract

**Objective:**

This study aimed to analyze cesarean section rates among women with GDM classified according to the Robson system and to identify independent factors associated with vaginal delivery.

**Method:**

A retrospective cohort study was conducted with pregnant women receiving care at the Hospital das Clínicas da Faculdade de Medicina da Universidade de São Paulo, between 2012 and 2020. A total of 1508 pregnant women with GDM were included and divided into two groups based on the mode of delivery. Statistical analyses were performed to assess differences between groups and identify predictive factors for vaginal delivery using Binary logistic regression.

**Results:**

The overall c-section rate was 65.3 %. Robson group 5 was the most representative, accounting for 39.53 % of all cesarean deliveries, with a single previous uterine scar being the primary indication (40.2 %). Spontaneous labor onset was the strongest independent predictor of vaginal delivery (OR = 8.601, 95 % CI 6.029‒12.270), whereas previous cesarean delivery (OR = 0.093, 95 % CI 0.065‒0.134), fetal distress (OR = 0.110, 95 % CI 0.071‒0.171), and multiple pregnancy (OR = 0.096, 95 % CI 0.036‒0.259) significantly reduced this likelihood.

**Conclusions:**

The high cesarean section rate among women with GDM, particularly in group 5, highlights the importance of previous uterine scar in determining the mode of delivery in this population. Encouraging spontaneous labor onset, provided that maternal and fetal conditions are adequately monitored, may be an effective strategy to reduce unnecessary cesarean sections in pregnancies complicated by GDM.

## Highlights

• Cesarean rates were highest in GDM women from group 5 with prior uterine scars.
• Spontaneous labor was the strongest predictor of vaginal delivery.
• Prior C-section, fetal distress, and multiples reduced vaginal delivery odds.
• Promoting safe spontaneous labor may lower C-sections in GDM cases.
• More prospective studies are needed to confirm findings and guide practice.

## Introduction

The prevalence of Gestational Diabetes Mellitus (GDM) varies between 1 % and 37.7 %,^1^ with a global mean of 16.2 %. In Brazil, the estimated prevalence is approximately 18 %.^1^ GDM is linked to unfavorable perinatal outcomes, including an elevated probability of cesarean delivery.

The increase in cesarean section rates represents a public health issue. Since 1985, the World Health Organization (WHO) has recommended a cesarean rate of 10 %–15 %^2^ to minimize maternal and neonatal mortality. In 2014, the WHO introduced the Robson classification as a universal framework for evaluating c-section rates. Latin America raises concern due to its notably high rates (42.8 %),^3^ surpassing those in regions such as Eastern Europe (25 %).^3^

Brazil has exceptionally high cesarean rates. A 2016 national study^4^ employing the Robson classification identified cesarean rates in high-risk pregnancies with substantial discrepancies between the public (67.7 %) and private (92.8 %) sectors. However, the study defined high-risk pregnancies broadly, without specifically isolating the impact of GDM on cesarean section rates.

A 2018 Australian cohort^5^ reported a c-section rate of 36.8 % among women with GDM, compared to 28.5 % in those without diabetes (*p* < 0.05). Prior cesarean deliveries were the primary predictor of cesarean sections, underscoring the necessity of reducing initial cesarean rates in nulliparous women. However, given Australia's lower overall cesarean rates compared to Brazil, these findings may not be entirely generalizable to the Brazilian context.

Due to the limited availability of data on this subject, this study aims to evaluate cesarean rates among women with GDM in Brazil using the Robson classification. Understanding the influence of GDM on cesarean rates will offer essential insights for clinical decision-making and health policy development.

## Objectives

This project aims to primarily analyze the rates and the main reasons for cesarean sections across different Robson groups in pregnant women with Gestational Diabetes Mellitus (GDM). Secondary objectives include evaluating independent predictors of vaginal delivery in women with GDM.

## Materials and methods

**Fig. 1:**
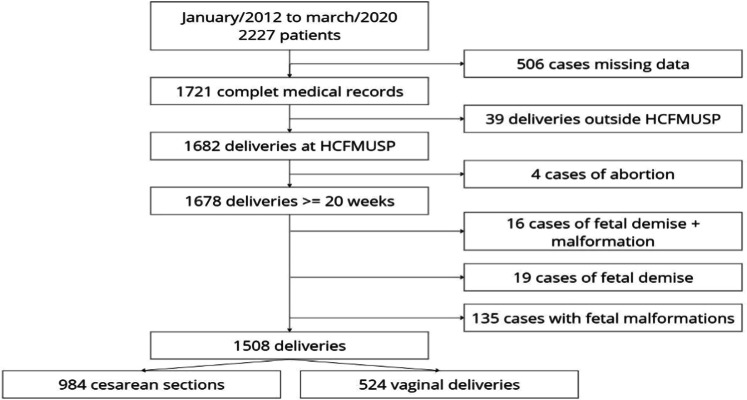
Patient selection flowchart for the study.

An observational retrospective cohort study was conducted, involving pregnant women who began prenatal care at a tertiary teaching hospital (Hospital das Clínicas, Faculdade de Medicina da Universidade de São Paulo) between January 2012 and March 2020. Eligibility criteria were the absence of pregestational diabetes or overt diabetes; diagnosis of GDM based on the criteria proposed by the International Association of Diabetes and Pregnancy Study Groups (IADPSG); Absence of fetal malformation and fetal dismiss; delivery performed at Hospital das Clínicas FMUSP. Given the retrospective nature of this study, no exclusion criteria were applied. Data were collected from the electronic records of pregnant women (Fig. 1).

Participants were divided based on the delivery outcome (mode of delivery) into two groups: vaginal delivery and cesarean section. These groups were compared based on sociodemographic and clinical variables.

Data were tabulated using Microsoft Excel software (2019) and analyzed using SPSS software (version 26).

The groups were described using qualitative data in terms of absolute and relative frequencies and compared using the Chi-Square test or Fisher's exact test, as appropriate. Quantitative data were described using measures of median and interquartile range and compared using the Mann-Whitney test. To assess the association between independent variables and the outcome of interest, a binary logistic regression analysis was performed using the Enter method. This approach allows the simultaneous inclusion of all variables deemed theoretically and clinically relevant, ensuring appropriate control for potential confounders. For model construction, the authors selected the variables that showed statistically significant differences between groups in the univariate analysis (*p* < 0.05). Variables remained in the model if they met the inclusion criterion of *p* < 0.05, and were considered for exclusion if *p* > 0.10, allowing refinement based on statistical contribution while maintaining the interpretability and integrity of the model. Data from the Oral Glucose Tolerance Test (OGTT) were not included in the analysis because a substantial proportion of participants ‒ approximately half ‒ had already been diagnosed with GDM based on early fasting blood glucose levels. Since these patients did not undergo the OGTT, including this variable would have significantly reduced the sample size available for regression analysis. Therefore, the authors chose to prioritize sample representativeness. A 95 % confidence level was adopted for the definition of statistical significance. There was no treatment of data and missing data were handled through complete-case analysis. The Model performance was assessed using the Receiver Operating Characteristic (ROC) curve (AUC), which evaluates the model’s discriminatory power.

Additionally, the two groups were subdivided according to the Robson classification into groups 1 to 10. For each category, the frequency of cesarean delivery and the main indications for cesarean were presented. This cohort is in agreement with the STROBE statement.

## Results

A total of 1508 cases were included, with 984 (65.3 %) cesarean deliveries and 524 (34.7 %) vaginal births. In the univariate analysis, cesarean deliveries were more common in older women, those with a higher BMI, a greater number of previous cesareans and pregnancies, multiple pregnancies, insulin use for GDM treatment, higher fasting glucose and OGTT levels, chronic hypertension, preeclampsia, fetal distress, and placenta previa. On the other hand, a higher number of previous deliveries, spontaneous onset of labor, and Premature Rupture of Membranes (PROM) were more frequent in the vaginal delivery group. Birth weight was similar between groups, but large-for-gestational-age babies were more common in cesareans (Table 1).

**Table 1:** Clinical and sociodemographic variables of pregnant women with gestational diabetes, according to the mode of delivery. (HCFMUSP, 2012‒2020).

	Cesarean		Vaginal		p-value
	N valid	Median [p25‒p75] n (%)	N valid	Median [p25‒p75] n (%)	
**Age (years)**	984	34 [30‒38]	524	32 [27‒36]	*<0.001*
**Height (m)**	895	1.60 [1.55‒1.65]	480	1.60 [1.56‒1.65]	0.802
**Color**	955		500		0.248
White		713 (74.7)		387 (77.4)	
Non-white		242 (25.3)		113 (22.6)	
**Marital status**	961		506		0.184
Single		294 (30.6)		172 (34)	
Married or stable union		667 (69.4)		334 (66)	
**Education**	856		449		0.980
< 9 years		293 (34.2)		154 (34.3)	
> 9 years		563 (65.8)		295 (65.7)	
**BMI (kg/m^2^)**^a^	965	29.3 [25.5‒34.3]	518	27.85 [24.3‒32.2]	*<0.001*
**Number of previous pregnancies**	984	2 [2‒4]	524	2 [1‒3]	*0.001*
**Number of previous deliveries**	984	1 [0‒2]	524	1 [0‒2]	*0.003*
**Number of previous cesareans**	984	1 [0‒1]	524	0 [0‒0]	*<0.001*
**Chronic hypertension**^a^	984	348 (35.4)	524	129 (24.6)	*<0.001*
**Multiple pregnancy**	984	58 (5.9)	524	8 (1.5)	*<0.001*
**Initial fasting blood glucose result (mg/dL)**	914	93 [84‒98]	496	91 [82‒96]	*<0.001*
**1st hour OGTT 75 *g* (mg/dL)**	439	164 [145‒188]	270	164 [138‒184]	*0.039*
**2nd hour OGTT 75 *g* (mg/dL)**	441	159 [135‒175]	274	156 [132‒170]	0.088
**GDM diagnosis**	984		524		*0.004*
Fasting		534 (54.3)		244 (46.6)	
OGTT 75 *g*		450 (45.7)		280 (53.4)	
**Gestational age at diagnosis (weeks)**	971	15 [13‒26]	518	19 [13‒26]	0.238
**GDM treatment**	982		523		*0.040*
Diet		755 (76.9)		426 (81.5)	
Diet + insulin		227 (23.1)		97 (18.5)	
**Preeclampsia**	984	175 (17.8)	524	57 (10.9)	*<0.001*
**Placenta previa**	984	18 (1.8)	524	1 (0.2)	*0.007*
**Spontaneous onset of labor**	984	147 (14.9)	524	282 (53.8)	*<0.001*
**PROM**^a^	984	164 (16.7)	524	141 (26.9)	*<0.001*
**Placental abruption**	984	7 (0.7)	524	1 (0.2)	0.275
**Fetal distress**	984	245 (24.9)	524	41 (7.8)	*<0.001*
**Gestational age at delivery (days)**	984	38.29 [39.29‒37]	523	38.71 [39.57‒37.29]	*<0.001*
**Preterm**	984	198 (20.1)	523	85 (16.3)	0.067
**Newborn gender**	983		523		0.202
Feminine		466 (47.4)		266 (50.9)	
Masculine		517 (52.6)		257 (49.1)	
**Birth weight (grams)**	983	3130 [3520‒2640]	523	3130 [3420‒2760]	0.479
**Fetal weight adequacy**	982		522		*0.013*
Appropriate for gestational age		773 (78.7)		439 (84.1)	
Small for gestational age		133 (13.5)		61 (11.7)	
Large for gestational age		76 (7.7)		22 (4.2)	
**Macrosomia**	983	44 (4.5)	534	15 (2.9)	0.124

a BMI, Body Mass Index; OGTT, Oral Glucose Tolerance Test; PROM, Premature Rupture of Membranes.

After logistic regression analysis, the strongest independent predictor of vaginal delivery was the spontaneous onset of labor (OR = 8.601, 95 % CI 6.029‒12.270). In contrast, previous cesarean delivery (OR = 0.093, 95 % CI 0.065‒0.134), fetal distress (OR = 0.110, 95 % CI 0.071‒0.171), and multiple pregnancy (OR = 0.096, 95 % CI 0.036‒0.259) significantly reduced the likelihood of vaginal birth. Conversely, a higher number of previous deliveries (OR = 1.548, 95 % CI 1.228‒1.952) and greater gestational age at delivery (OR = 1.013, 95 % CI 1.002‒1.025) were associated with an increased probability of vaginal delivery. The Receiver Operating Characteristic (ROC) curve of the model showed an Area Under the Curve (AUC) of 0.87 (95 % CI 0.874‒0.909) (Table 2).

**Table 2:** Logistic regression for identifying independent factors associated with vaginal delivery in pregnant women with gestational diabetes (HCFMUSP, 2012‒2020).

	OR	95 % IC
**Age**	0.946	*0.922‒0.971*
**BMI**^a^	1.001	0.975‒1.027
**Number of previous pregnancies**	0.992	0*.*830‒1.186
**Number of previous deliveries**	1.548	*1.228‒1.952*
**Number of previous cesareans**	0.093	*0.065‒0.134*
**Chronic hypertension**	0.854	0.589‒1.239
**Multiple pregnancy**	0.096	*0.036‒0.259*
**Initial fasting blood glucose result (mg/dL)**	1.001	0.979‒1.023
**GDM diagnosis by OGTT**^a^	1.456	0.894‒2.373
**GDM treatment (diet + insulin)**	0.835	0.567‒1.230
**Preeclampsia**	1.234	0.777‒1.960
**PROM**^a^	1.416	0.978‒2.051
**Placenta previa**	0.228	0.026‒2.014
**Fetal distress**	0.110	*0.071‒0.171*
**Spontaneous onset of labor**	8.601	*6.029‒12.270*
**Gestational age at delivery (days)**	1.013	*1.002‒1.025*
**Newborn small for gestational age**^b^	1.317	0.843‒2.057
**Newborn large for gestational age**^b^	0.550	0.283‒1.069
**Constant**	0.095	

a BMI, Body Mass Index; OGTT, Oral Glucose Tolerance Test; PROM, Premature Rupture of Membranes.

b The reference used for analysis are pregnancies with newborns Appropriate for Gestational Age (AGA). Variables included in Step 1: Age, BMI, Number of previous pregnancies, Number of previous deliveries, Number of previous cesareans, Initial fasting blood glucose result, Gestational age at delivery, Preeclampsia, PROM, Hypertension, GDM diagnosis by OGTT, Fetal weight adequacy, Spontaneous labor onset, Multiple pregnancy, Placenta previa, Fetal distress, GDM treatment.

Regarding cesarean rates, lower numbers were observed in groups 1‒4, while higher rates were found in groups 5‒10, reaching 95.1 % in group 7 (Table 3). Additionally, in terms of each group's contribution to the total number of cesarean deliveries, group 5 was the most representative, accounting for 39.53 % of all cesareans.

**Table 3:** Cesarean rates in pregnant women with gestational diabetes according to Robson Groups (HCFMUSP, 2017‒2020).

Robson Group	Total	Cesarean	Vaginal	C-section frequency within group	% of the group in the total number of c-sections	% of the group in the total number of deliveries
	n	n	n	%	%	%
1	100	33	67	33.0	3.35	6.6
2	285	188	97	66.0	19.11	18.9
3	126	21	105	16.7	2.13	8.4
4	201	78	123	38.8	7.93	13.3
5	431	389	42	90.3	39.53	28.6
6	27	25	2	92.6	2.54	1.8
7	41	39	2	95.1	3.96	2.7
8	66	58	8	87.9	5.89	4.4
9	13	12	1	92.3	1.22	0.9
10	218	141	77	64.7	14.33	14.5
Total	1508	984	524	65.3	100	100

**Fig. 2:**
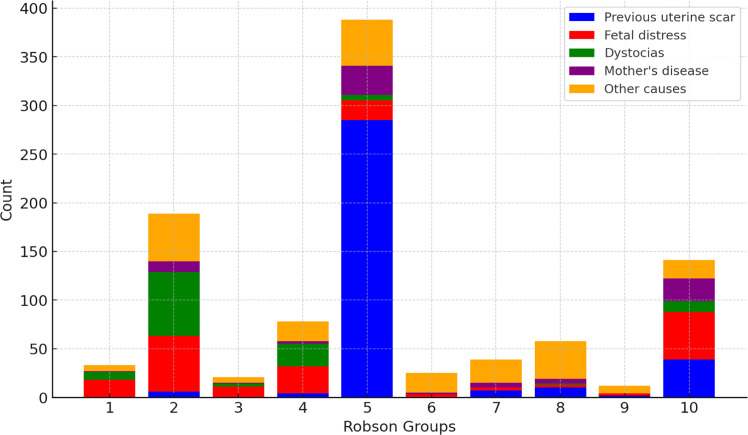
Primary indications for cesarean section in pregnant women with gestational diabetes according to Robson Classification (HCFMUSP, 2017‒2020). *Mother’s disease: decompensation of pre-existing maternal conditions and pathologies that contraindicate vaginal delivery. Other causes include placenta previa, cephalopelvic disproportion, previous tumor, placental abruption, pelvic abnormality, cord prolapse, suspected macrosomia, alloimmunization, multiple gestation, breech presentation and maternal desire.

Regarding the overall primary indications for cesarean, a previous uterine scar accounted for 35.9 %, followed by fetal distress at 19.7 %, dystocia at 12 %, and maternal causes at 8.2 %. Other causes contributed to 24.1 % of the indications. This study showed a notable high prevalence of dystocia in groups 4 (29.5 %) and 2 (34.9 %), with dystocia being the leading indication in the latter group. Fetal distress was the primary indication in groups 1 (56.3 %), 3 (52.4 %), 4 (35.4 %), and 10 (34.7 %). In group 2, other significant causes included cephalopelvic disproportion (10 %) and suspected macrosomia (2.6 %). Similarly, group 4 also exhibited suspected macrosomia (6.4 %) and cephalopelvic disproportion (5.1 %) as prevalent indications for c-section. Group 5 stood out, where a previous uterine scar was the primary indication for cesarean delivery in 73.5 % of cases. A single previous uterine scar accounted for 40.2 %. In groups 6, 7, 8 and 9, 'other causes' were the most prevalent indication, mainly abnormal presentation and multiple gestation. In group 10, a previous uterine scar was the second most prevalent cause, accounting for 27.7 % of indications, followed by maternal causes at 16.3 %. Fig. 2 provides a visual representation of the primary indications for cesarean delivery across these groups.

## Discussion

This cohort study involving 1508 patients with Gestational Diabetes Mellitus (GDM) found a cesarean section rate of 65.3 %. The primary indications for cesarean delivery were a previous uterine scar (35.5 %) and fetal distress (17.8 %). Independent predictors for higher rates of vaginal delivery included a higher number of previous deliveries and spontaneous onset of labor. Conversely, advanced maternal age, the number of previous cesarean sections, multiple pregnancies, and fetal distress were associated with lower rates of vaginal delivery.

The cesarean section rates in women with GDM in this study (65.3 %) were notably higher than those reported in a cohort from New South Wales,^5^ Australia, which had a rate of 36.8 %. Comparing the cesarean rates among women with GDM to the overall cesarean rates in the Australian (35.6 %) and Brazilian (51.9 %) populations, the authors observed a more pronounced relative increase in cesarean rates among women with GDM in Brazil. While in Australia, this increase was approximately 3 %, in our analysis, it was around 26 %. These findings highlight the significant impact of GDM on cesarean rates, especially in Brazil, where cesarean rates are already very high. The cesarean rate observed in this study aligns with data from Nakamura et al. for high-risk pregnancies in the Brazilian public sector (67.7 %), reflecting the trend of higher cesarean rates in more complex obstetric contexts.

Comparing our findings with an ecological study conducted in Brazil by Pereira VB et al.,^6^ cesarean section rates in both datasets show some notable similarities and differences. In groups 1, 2, 3, 6, 7, and 8, the rates are quite similar, reflecting a consistent trend between Pereira´s data, which assessed the general population, and ours. However, in group 5, the cesarean section rate in our analyses (90.3 %) is slightly higher than theirs (85.9 %). Additionally, in group 10, including preterm births, the cesarean section rate in our sample (64.7 %) was significantly higher than the general population data (54.0 %).^6^

Regarding the difference found in group 10, it can be partially explained by the fact that most preterm births in our study were elective rather than spontaneous. Unlike the general population, where a larger proportion of preterm deliveries occur spontaneously, the elective nature of these cases points to the presence of maternal or fetal indications for preterm delivery, which increases the likelihood of cesarean section.^7^ It is important to notice the risk of bias for confounding variables. GDM patients commonly have other comorbidities, such as hypertension and preeclampsia. In this study, the prevalence rates were 31.6 % and 15.4 %, respectively. As a result, this increases the likelihood of fetal distress, which was the main indication in this group (34.7 %). Within the same group, maternal causes also stood out, accounting for 16.3 % of the primary indications. These figures emphasize the importance of effective maternal monitoring and the management of comorbidities to reduce cesarean rates in this group.

Group 5 was the largest group (28.6 %) and also the most representative, accounting for almost 40 % of all cesarean deliveries. This high proportion of Group 5 emphasizes the alarming rates of cesarean in Brazil during the last decades. Culturally, cesarean births in Brazil have been associated with higher social status and are perceived as a safer option. Additionally, there is a widespread belief that vaginal births are more painful and risky, whereas cesarean sections are perceived as a more controlled and convenient option. Besides, current policies of the Brazilian private healthcare system further encourage the high cesarean rates, as doctors are paid per procedure, incentivizing cesareans, which are more profitable, schedulable and less time-consuming. Despite recent public policies aimed at reducing cesarean rates, such as increased reimbursement for vaginal deliveries, these measures still have had limited success due to entrenched medical practices and patient preferences.

Still in regard to Group 5, the cesarean rate was particularly high (90.3 %), with 40.2 % of cases having a single previous uterine scar as the primary indication for a new cesarean.

Patients with a previous cesarean section have the option to pursue a Trial of Labor After Cesarean (TOLAC) or a Planned Repeat Cesarean Birth (PRCB). Vaginal Birth After Cesarean (VBAC) avoids potential adverse outcomes associated with cesarean deliveries. However, TOLAC carries risks such as uterine rupture, whereas PRCB avoids these risks as well as other potential complications associated with intrapartum cesarean birth. Besides clinical variables, patient preferences, and possible complications, the decision should consider the availability of TOLAC, as ACOG^9^ recommends facilities capable of performing an emergency cesarean birth. When resources are limited, risks should be discussed with the patient, who may choose a facility with better resources, accept higher risks for TOLAC at their preferred facility, or opt for PRCB.

Moreover, although women with two prior low transverse uterine incisions are considered candidates for TOLAC,^9^ they have a slightly higher prevalence of uterine rupture compared to one previous c-section (1.59 % vs. 0.72 %).^10^ In contrast, patients with prior vertical cesarean incisions or a history of uterine rupture are generally not candidates for TOLAC due to a higher risk of uterine rupture. Of notice, uterine rupture is two to three times more frequent when labor is induced (0.87 % with prostaglandin induction, and 0.29 % with Foley catheter induction combined with amniotomy) compared to spontaneous VBAC (0.15 %).^11^ While GDM is not considered a contraindication to TOLAC, it is important to consider potential risks such as shoulder dystocia, particularly in cases of macrosomia. However, there is no specific guidance for TOLAC in GDM patients. These data emphasize the importance of carefully selecting the mode of delivery and induction method, considering maternal and fetal risks, and the need for further studies regarding TOLAC in GDM patients.

Encouraging spontaneous labor was identified as a key factor in increasing the likelihood of vaginal delivery, particularly for nulliparous women, where a previous uterine scar is not a risk factor. Spontaneous labor increases the chances of vaginal delivery by 8.6 times. On the other hand, each previous cesarean significantly reduced the chances of a vaginal delivery by more than 90 %. These findings suggest that promoting spontaneous labor could be an effective strategy to reduce cesarean rates, especially in nulliparous patients. However, it is essential to ensure that clinical conditions and fetal well-being are properly assessed to guarantee the safety of both the mother and the baby. Notably, in group 2, a high prevalence of dystocia was observed, which may be linked to early labor induction rather than allowing spontaneous onset when conditions permit. This approach could potentially reduce unnecessary cesarean deliveries. In light of current national and international guidelines,^8^^,^^12^ it is important to note that women diagnosed with gestational diabetes managed through diet alone (A1GDM) should not undergo delivery prior to 39 weeks of gestation unless specific clinical indications ‒ such as signs of fetal compromise ‒ justify earlier intervention. For this group, it is generally appropriate to continue pregnancy up to 40 weeks and 6 days, accompanied by appropriate fetal monitoring. In contrast, for individuals requiring pharmacological treatment for gestational diabetes (A2GDM), delivery is advised between 39 weeks and 39 weeks and 6 days. These recommendations, in conjunction with the findings of our study, reinforce the notion that avoiding non-medically indicated early labor induction may contribute to lowering cesarean delivery rates, including the critical Robson Group 5. Therefore, national guidelines for Gestational Diabetes Care must encourage not anticipating the delivery if maternal and fetal conditions are reassuring.

This study has some limitations, including its retrospective, single-center design, which limits the generalizability of the findings to other populations. Furthermore, since it was conducted in a tertiary hospital where women with multiple comorbidities are more common, there may be selection bias. Consequently, although conducting the study in a high-complexity tertiary referral center ensures a standardized approach to care, this setting may not reflect the diversity of clinical practices, resource availability, and patient profiles found in community or private hospitals. In lower-complexity settings, cesarean rates and the factors influencing delivery mode may differ due to variations in institutional protocols, provider experience, and access to emergency services. Future studies should assess whether these findings hold true across different levels of healthcare. Nevertheless, the sample size of 1508 patients provides a strong foundation for the findings of a single-center study.

Considering the high prevalence of gestational diabetes in Brazil and the scarcity of national data on the topic, the findings of this study are highly relevant, offering significant contributions to understanding the main indications and predictors of cesarean delivery in this population. Identifying these factors is crucial for optimizing the management of these patients to reduce cesarean section rates, a persistent challenge in Brazilian obstetric healthcare. Although the results provide valuable and clinically applicable insights, additional prospective and multicenter studies are needed to further explore the best treatment and follow-up protocols for this specific population. Such studies are essential for implementing effective strategies aimed at reducing the high cesarean rates in Brazil, ultimately improving perinatal and maternal outcomes.

## Conclusion

This study demonstrated a substantially high cesarean section rate among women with GDM, with Robson group 5 being the most prominent, where a previous uterine scar was the leading indication for cesarean delivery. Furthermore, multivariate analysis identified spontaneous labor onset as the strongest predictor of vaginal delivery, while previous cesarean section, fetal distress, and multiple pregnancies significantly reduced this likelihood.

These findings highlight the importance of implementing strategies to promote vaginal delivery whenever clinically safe, particularly by avoiding unnecessary early labor inductions and allowing patients in group 5 to enter spontaneous labor whenever possible. However, it is important to acknowledge that spontaneous labor may not always be feasible or safe, particularly in high-risk pregnancies. Therefore, the approach to labor should be individualized, considering the unique circumstances and risks of each patient. Encouraging spontaneous labor onset, along with careful maternal and fetal assessment, may help reduce cesarean rates in women with GDM.

Although this study provides valuable insights, further prospective, multicenter research is needed to validate these findings and examine the long-term outcomes of different delivery modes in women with GDM. Such studies would address the limitations of the current research and enhance the evidence base for clinical practice.

## Ethical considerations

The study was conducted with the approval of the Ethics Committee of Hospital das Clínicas FMUSP, São Paulo, Brazil (CAAE 48,868,915.9.0000.0068). Informed consent was waived due to the retrospective nature of the study. All methods were carried out in accordance with relevant guidelines and regulations.

## CRediT authorship contribution statement

**Ana Paula Veiga de Oliveira:** Data curation, Validation, Writing – original draft. **Tatiana Assunção Zaccara:** Data curation, Validation, Writing – review & editing. **Marcela Del Carlo Bernardi:** Data curation, Validation, Writing – review & editing. **Cristiane de Freitas Paganoti:** Data curation, Validation, Writing – review & editing. **Fernanda Cristina Ferreira Mikami:** Data curation, Validation, Writing – review & editing. **Rossana Pulcineli Vieira Francisco:** Data curation, Validation, Writing – review & editing. **Rafaela Alkmin da Costa:** Conceptualization, Formal analysis, Supervision, Writing – review & editing.

## Conflicts of interest

The authors declare no conflicts of interest. Although this study was supported by a scientific scholarship provided to the author APVO by the Fundação de Amparo à Pesquisa do Estado de São Paulo (FAPESP) (Process number: 2023/09,560–0), the funder did not have a specific role in the conceptualization, design, data collection, analysis, decision to publish, or preparation of the manuscript.
